# Adversarial Attacks against Deep-Learning-Based Automatic Dependent Surveillance-Broadcast Unsupervised Anomaly Detection Models in the Context of Air Traffic Management

**DOI:** 10.3390/s24113584

**Published:** 2024-06-02

**Authors:** Peng Luo, Buhong Wang, Jiwei Tian, Chao Liu, Yong Yang

**Affiliations:** School of Information and Navigation, Air Force Engineering University, Xi’an 710051, China

**Keywords:** air traffic management, ADS-B, anomaly detection, deep learning, adversarial attacks

## Abstract

Deep learning has shown significant advantages in Automatic Dependent Surveillance-Broadcast (ADS-B) anomaly detection, but it is known for its susceptibility to adversarial examples which make anomaly detection models non-robust. In this study, we propose **T**ime **N**eighborhood **A**ccumulation **I**teration **F**ast **G**radient **S**ign **M**ethod (**TNAI-FGSM**) adversarial attacks which fully take into account the temporal correlation of an ADS-B time series, stabilize the update directions of adversarial samples, and escape from poor local optimum during the process of iterating. The experimental results show that TNAI-FGSM adversarial attacks can successfully attack ADS-B anomaly detection models and improve the transferability of ADS-B adversarial examples. Moreover, the TNAI-FGSM is superior to two well-known adversarial attacks called the Fast Gradient Sign Method (FGSM) and Basic Iterative Method (BIM). To the best of our understanding, we demonstrate, for the first time, the vulnerability of deep-learning-based ADS-B time series unsupervised anomaly detection models to adversarial examples, which is a crucial step in safety-critical and cost-critical Air Traffic Management (ATM).

## 1. Introduction

As a new generation of air traffic management surveillance technology, ADS-B can broadcast aircraft position, velocity, heading and other flight information in real-time which improves the visibility of air traffic, reduces flight conflicts and enhances the efficiency and safety of ATM [1]. However, since ADS-B data are broadcast in plaintext format and lack an authentication mechanism, the attackers can easily jam, eavesdrop, modify, inject and delete ADS-B messages [2,3,4]. Therefore, it is crucial to detect anomaly data caused by ADS-B attacks [5,6].

Compared to other methods such as encryption, physical layer information and multilateration, deep learning has demonstrated tremendous success in the field of detecting ADS-B anomaly data [7,8,9,10]. The deep learning method usually detects ADS-B anomaly data based on prediction errors and reconstruction errors. Taking into account the temporal correlation of ADS-B data, Long Short-Term Memory (LSTM) has been utilized for detecting ADS-B anomaly data by analyzing prediction errors [11]. In order to improve the accuracy of anomaly detection, an LSTM-Encoder-Decoder is used to reconstruct ADS-B data and determine whether ADS-B data are anomalous [12]. Considering the maneuvering characteristic of ADS-B flight data, a Variational Autoencoder (VAE) and Gate Recurrent Unit (GRU) model are used to detect ADS-B anomaly data on the basis of reconstruction errors [13]. The deep learning method makes full use of the temporal correlation of ADS-B data to establish anomaly detection models, which have the advantage of detecting ADS-B anomaly data accurately and quickly.

However, deep learning itself can be easily fooled by adversarial examples [14,15]. Therefore, deep-learning-based ADS-B anomaly detection models could also be attacked by adversarial examples. The applications of ADS-B anomaly detection models end with decision making by pilots or onboard automation systems [16]. Therefore, it may result in serious consequences such as flight deviation, flight delays and aircraft collisions once deep-learning-based ADS-B anomaly detection models are attacked by adversarial examples [17]. Not only the accuracy, but also the robustness of ADS-B anomaly detection models need to be investigated. At present, adversarial examples have been widely studied in computer vision fields such as image classification, object detection and face recognition [18,19,20]. From the perspective of image classification, adversarial examples refer to modifying the original image with small, imperceptible adversarial perturbations, causing the modified image to be classified incorrectly [21,22]. However, the application of adversarial samples in non-image fields, especially in time series prediction and regression problems, is vastly limited [23]. This also includes the lack of research on adversarial samples for ADS-B time series anomaly detection despite the increasing success of deep learning in the field of ADS-B time series anomaly detection. In our previous work, we managed to craft adversarial examples to fool an ADS-B spoofing detection classifier based on a Manchester Encoding Attack (MEA) [24]. The biggest flaw of this work is that it assumes that the victim model is supervised and considers ADS-B spoofing detection as a classification problem. However, there is scarce labeled ADS-B anomaly data in the real-world environment [25]. Therefore, the victim ADS-B anomaly detection model should be considered as an unsupervised regression problem that makes full use of the temporal correlation in an ADS-B time series. There are two reasons why previous methods, including FGSM and BIM, are not suitable for adversarial attacks against ADS-B anomaly detection models [14,15]. First, previous methods failed to take into account the temporal correlation of an ADS-B time series, thus resulting in a poor success rate of adversarial attacks. Second, previous adversarial attack methods failed to stabilize the update directions of adversarial samples and could not escape from poor local optimum during the process of iterating, thus leading to poor transferability of adversarial attacks.

In order to solve the problems mentioned above, this paper proposes a TNAI-FGSM to craft imperceptible adversarial samples to fool the victim deep-learning-based ADS-B time series unsupervised anomaly detection models. The main contributions of this paper are summarized as follows:

(I) The TNAI-FGSM is proposed to craft ADS-B adversarial samples. To the best of our understanding, we demonstrate, for the first time, the vulnerability of deep-learning-based ADS-B unsupervised anomaly detection models to adversarial attacks.

(II) Based on a time neighborhood mechanism, the TNAI-FGSM fully takes into account the temporal correlation of an ADS-B time series which can improve the success rate of adversarial attacks.

(III) Based on an accumulation iteration mechanism, the TNAI-FGSM can stabilize the update directions of adversarial samples and escape from poor local optimum during the process of iterating, thus improving the transferability of adversarial attacks.

(IV) The experimental results show that the TNAI-FGSM is superior to other adversarial attack methods in terms of success rate, stealthiness and transferability.

The rest of this paper is organized as follows. Section 2 introduces the basic knowledge about security solutions to ADS-B data, including deep learning for ADS-B anomaly detection, and briefly discusses adversarial attacks. Section 3 formalizes the adversarial attacks against deep-learning-based ADS-B unsupervised anomaly detection models and explains the FGSM, BIM and our proposed TNAI-FGSM for crafting adversarial samples. Section 4 shows the experimental results of adversarial attacks against four types of ADS-B anomaly detection models. The transferability property of adversarial attacks is evaluated. We also use adversarial training to defend against ADS-B adversarial attacks in this section. Section 5 provides a discussion about the limitations of this work and future work. Section 6 makes conclusions.

## 2. Related Works

### 2.1. ADS-B

**Figure 1:**
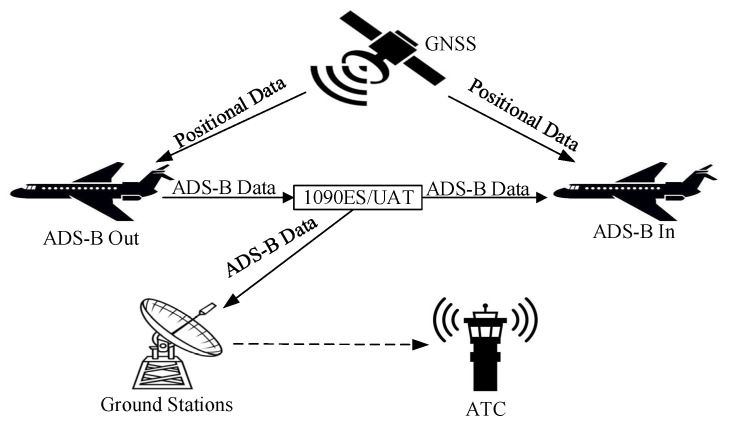
General overview of ADS-B.

ADS-B has become a core technology for the new generation ATM system. ADS-B has the advantages of wide coverage, high accuracy, low cost and support for information sharing. Figure 1 gives a general overview of an ADS-B system. Aircraft obtain precise position, heading and velocity information from the Global Navigation Satellite System (GNSS) and on-board devices. Aircraft equipped with ADS-B Out broadcast ADS-B data over the 1090 MHz Extended Squitter (1090ES) or Universal Access Transceiver (UAT) communication channel. Nearby aircraft equipped with ADS-B In can receive ADS-B data. The ground stations receive and process ADS-B data which are then sent to the Air Traffic Control (ATC) system. However, since ADS-B data are broadcast in plaintext format and lack an authentication mechanism, attackers can easily jam, eavesdrop, modify, inject and delete ADS-B messages [26].

### 2.2. Secure Solutions to ADS-B Data

At present, security solutions for ADS-B risks mainly include four categories. The four categories are encryption, physical layer information, multilateration and deep-learning-based ADS-B anomaly detection. The first three categories can be called traditional methods. Table 1 provides a comparison of the existing security solutions. In this paper, we focus on deep-learning-based ADS-B unsupervised anomaly detection models as the victim models. Therefore, in the following, we present less traditional methods and analyze more deep-learning-based ADS-B anomaly detection models.

#### 2.2.1. Traditional Methods

(1) **Encryption:** An ADS-B encryption method uses keys to directly encrypt ADS-B plaintext or generate corresponding integrity information to provide an information basis for ADS-B security reinforcement [7,27,28]. The encryption method needs to modify the original ADS-B protocol, which hinders system compatibility and international interoperability.

(2) **Physical layer information:** When ADS-B data are transmitted by wireless communication, the data are equipped with physical fingerprint information, which is the basis for analyzing the probability of ADS-B anomaly data [8,29]. However, when attackers obtain prior knowledge of physical layer information through statistical analysis, the applicability of the method will need further verification for sophisticated ADS-B data attacks.

(3) **Multilateration:** The multilateration method usually uses Time Difference of Arrival (TDOA) to measure the position of the aircraft and compares it with the parsed ADS-B position [9,30]. If the difference between the two positions is too large, the ADS-B message is illegal. However, the multilateration method requires deploying multiple ground stations, which is not economical.

#### 2.2.2. Deep Learning for ADS-B Anomaly Detection

Recently, deep learning has demonstrated tremendous success in the field of detecting ADS-B anomaly data [2,3,10]. ADS-B data have the characteristics of temporal correlation, maneuvering characteristics of ADS-B flight data, the lack of labeled ADS-B anomaly data in real environments and the characteristic of multidimensional attributes such as longitude, latitude, altitude, velocity and heading at any moment [17]. To achieve excellent performance, the deep learning method needs to take into account the above characteristics of ADS-B data. The deep learning method usually detects ADS-B anomaly data based on prediction errors and reconstruction errors [31]. Based on fully considering the characteristic of temporal correlation and the lack of labeled ADS-B anomaly data in real environments, LSTM and GRU are usually used to establish unsupervised ADS-B anomaly detection models and determine whether ADS-B data are anomalous based on prediction errors [11]. In order to improve the accuracy of anomaly detection, an LSTM-Encoder-Decoder is used to reconstruct ADS-B data and determine whether ADS-B data are anomalous based on reconstruction errors [12]. In order to fully consider the maneuvering characteristics of ADS-B flight data, a VAE is used to detect ADS-B anomaly data by analyzing reconstruction errors [13]. The LSTM-Encoder-Decoder and VAE also use LSTM and Gated Recurrent Unit (GRU) as the hidden layer to preserve the temporal correlation of ADS-B data [32]. In this paper, we focus on four representative deep-learning-based ADS-B unsupervised anomaly detection models as the victim models which are based on the VAE, LSTM and LSTM-Encoder-Decoder. The specific descriptions of the four victim models are shown in Section 3.2: Typical ADS-B training module.

### 2.3. Adversarial Attacks

Adversarial attacks are proposed for image recognition at first [14,33]. From the perspective of image recognition, adversarial examples refer to modifying the original image with small, imperceptible adversarial perturbations, causing the modified image to be classified incorrectly with high confidence [21,34]. Based on the idea above, some adversarial attack algorithms have been proposed, among which the most classic algorithms are the FGSM and BIM [14,15,35]. By utilizing the gradient information of the loss function, the FGSM generates adversarial examples that are designed to mislead the model’s prediction or classification [17,35]. The BIM is an extension of the FGSM. The BIM performs multiple small perturbations along the direction of an increasing gradient through iterative methods, while the FGSM only performs one step of perturbations [15,36]. Although the application of adversarial samples in image fields is becoming more and more popular, studies on adversarial samples in image fields, especially in time series prediction and regression problems, are vastly limited [23]. This also includes the lack of research on adversarial samples for ADS-B time series anomaly detection despite the increasing success of deep-learning-based ADS-B time series anomaly detection. In our previous work, we managed to craft adversarial examples to fool an ADS-B spoofing detection classifier based on a Manchester Encoding Attack (MEA) [24]. The biggest flaw of this work is that it assumes that the victim model is supervised and assumes ADS-B spoofing detection is a classification problem. However, there is scarce labeled ADS-B anomaly data in a real-world environment [25]. Therefore, the victim ADS-B anomaly detection model should be considered as an unsupervised regression problem that makes full use of the temporal correlation in ADS-B time series. There are two reasons why previous methods, including the FGSM and BIM, are not suitable for adversarial attacks against ADS-B anomaly detection models [14,15,35,36]. First, previous methods failed to take into account the temporal correlation of an ADS-B time series, thus resulting in a poor success rate of adversarial attacks. Second, previous adversarial attack methods failed to stabilize the update directions of adversarial samples and could not escape from poor local optimum during the process of iterating, thus leading to poor transferability of adversarial attacks. Table 2 summarizes the gaps of previous adversarial attack methods when they are considered to be applied to deep-learning-based ADS-B time series unsupervised anomaly detection models.

## 3. Adversarial Attacks against ADS-B Anomaly Detection Models

In this section, we formalize adversarial attacks against deep-learning-based ADS-B unsupervised anomaly detection models and explain our proposed TNAI-FGSM for crafting adversarial samples.

### 3.1. Formalization of Adversarial Attacks

**Definition** **1.** *Let* $X=[X_{1},X_{2},\ldots ,X_{t},\ldots ,X_{M}]$  *be the original ADS-B multivariate time series received by ADS-B anomaly detection models.* M=|X|  *is the length of* X. $X_{t}$ *is an N-dimensional vector at time* t *where* t∈[1,M]*. Each dimension of*  $X_{t}$ *represents the ADS-B feature, which includes latitude, longitude, altitude, velocity and heading.*

**Definition** **2.** *Let* $f(•):R^{N\times M}\to F$ *represent a deep-learning-based ADS-B unsupervised anomaly detection model. This task can be represented as training a deep learning model in order to predict F from the input*$X=[X_{1},X_{2},\ldots ,X_{t},\ldots ,X_{M}]$*. F can be normal or an anomaly.*

**Definition** **3.** *Let* $X^{adv}=X+\eta$ *denote the attacked ADS-B time series.* η *is a small, imperceptible noise.* $X^{adv}$ *is a perturbed version of* X *with the aim of* F’≠F *and* $\left\|X^{adv}-X\right\|\leq \varepsilon$*, where* f(X)=F*,* $f(X^{adv})=F’$ *and* ε≥0 *is a very small magnitude. Therefore, we give the formalization of adversarial attacks against deep-learning-based ADS-B unsupervised anomaly detection models. Given a trained anomaly detection model f and original input ADS-B time series* X*, crafting adversarial examples* $X^{adv}$ *can be represented as an optimization problem with constraints as follows:*

(1) $$\begin{array}{c} \underset{X^{adv}}{\min}\left\|X^{adv}-X\right\|\\ s.t.\;f(X)=F,\;f(X^{adv})=F’,\;F\neq F’ \end{array}$$

**Figure 2:**
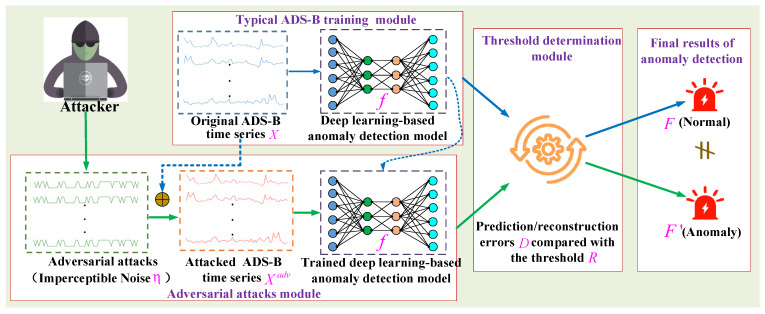
General architecture of adversarial attacks against an ADS-B anomaly detection model.

$\eta =X^{adv}-X$ is a small, imperceptible noise added to X. Figure 2 also shows the general architecture of adversarial attacks against ADS-B anomaly detection models. Below, we separately demonstrate a typical ADS-B training module, threshold determination module and adversarial attacks module as shown in Figure 2.

### 3.2. Typical ADS-B Training Module

#### 3.2.1. Selection Criteria

In this work, we focus on four representative deep-learning-based ADS-B unsupervised anomaly detection models as the victim models which are based on a VAE, LSTM, GRU and LSTM-Encoder-Decoder [11,12,13]. The reasons for selecting these four victim models as targets include the following.

(1) In terms of scope, the four selected models include both types of unsupervised ADS-B anomaly detection methods, which are based on prediction errors and reconstruction errors.

(2) In terms of classic and state-of-the-art methods, models based on LSTM, GRU and LSTM-Encoder-Decoder are representative because later ADS-B anomaly detection methods are generally improved based on the four models to improve the accuracy of anomaly detection. Furthermore, an ADS-B anomaly detection model based on a VAE is relatively state-of-the-art and fully considers the maneuvering characteristic of ADS-B flight data.

(3) In fact, not only the four ADS-B anomaly detection models mentioned above but also all the other existing models have not considered adversarial attacks. We can reasonably infer that all other existing ADS-B anomaly detection models are vulnerable to adversarial attacks based on the transferability property of adversarial samples.

#### 3.2.2. Selected ADS-B Anomaly Detection Baselines

We selected the following victim ADS-B anomaly detection models based on the above criteria. **(1) ADS-B anomaly detection based on LSTM and GRU** [11]**:** The core idea of an LSTM-based and GRU-based method is prediction errors. Specifically, the neural network composed of LSTM and GRUs is used for ADS-B predictive training. The threshold is set by calculating the difference value between the ADS-B predicted value and ADS-B actual value. If the difference value is greater than the threshold, the ADS-B data are an anomaly, and vice versa. **(2) ADS-B anomaly detection based on an LSTM-Encoder-Decoder and VAE** [12,13]**:** An LSTM-Encoder-Decoder and VAE detect ADS-B anomaly data based on reconstruction errors. In order to improve the accuracy of anomaly detection, an LSTM-Encoder-Decoder is used to reconstruct ADS-B data and determine whether ADS-B data are anomalous. In order to fully consider maneuvering characteristics of ADS-B flight data, a VAE is used to detect ADS-B anomaly data on the basis of reconstruction errors. An LSTM-Encoder-Decoder and VAE also use LSTM and GRUs as the hidden layer to preserve the temporal correlation of ADS-B data. The parameter settings of the four selected models are basically consistent with those in reference [11,12,13]. In order to solve the problem of poor adaptability of the threshold determined by manually analyzing the prediction/reconstruction errors, Support Vector Data Description (SVDD) is used to train the difference value [37].

### 3.3. Threshold Determination Module

This module compares prediction/reconstruction errors with the threshold of anomaly detection. If prediction/reconstruction errors are greater than the threshold, the ADS-B data are an anomaly, and vice versa. SVDD can solve the threshold adaptive problem and achieve the relatively optimal accuracy of ADS-B anomaly detection, so it is selected for determining the threshold [13,37]. The difference value between the predicted/reconstructed value and the actual value is put into SVDD for training, and then the threshold of anomaly detection can be obtained. SVDD can be expressed as the following optimization problem:

(2) $$\begin{array}{ll} & \min H(R,a)=R^{2}+C\sum_{j}^{n}\xi_{j}\\ s.t. & \left\{ \begin{array}{l} {\left\|D_{j}-a\right\|}^{2}\leq R^{2}+\xi_{j},\;(j=1,2,\ldots ,L)\\ \xi_{j}\geq 0 \end{array}\right. \end{array}$$

R is the radius of SVDD hypersphere, which is the threshold of anomaly detection. a is the center of the hypersphere. $\xi_{j}$ is the slack variable. C is the penalty coefficient which is used to control the volume of the hypersphere, and the value of C is usually 1. If the distance from $D_{j}$ to the center a is denoted as $S(D_{j})$, then $S(D_{j})$ can be expressed as follows:

(3) $$S(D_{j})=\left\|D_{j}-a\right\|=\sqrt{(D_{j},D_{j})-2\sum_{i=1}^{L}\lambda_{i}(D_{i},D_{j})+\sum_{i=1}^{L}\sum_{k}^{L}\lambda_{i}\lambda_{k}(D_{i},D_{k})}$$

It means that the sample $D_{j}$ are anomaly data when $S(D_{j})>R$. The sample $D_{j}$ are normal data when $S(D_{j})\leq R$.

### 3.4. Adversarial Attacks Module

In the adversarial attacks module, we first define and analyze adversarial attackers from three aspects which are capability, knowledge and goals. Then, we explain the FGSM, BIM and our proposed TNAI-FGSM for crafting ADS-B adversarial samples.

#### 3.4.1. Definition of the Adversarial Attackers

**Capabilities:** We consider adversarial attackers whose objective is to reduce the effectiveness of the victim ADS-B anomaly detection models. Adversarial attackers could appear in the ground station, air traffic control center, airlines and aircraft. Adversarial attackers with Software Defined Radio (SDR) devices can compromise the ADS-B sensors and 1090 MHz Extended Squitter (1090ES) or Universal Access Transceiver (UAT) communication channel [4,5]. Based on the capabilities above, attackers can apply the adversarial samples by modifying ADS-B data.

**Knowledge:** To verify the vulnerability of ADS-B anomaly detection models, we launch a white-box attack and black-box attack, respectively. (1) ADS-B white-box attackers know all the information and parameters inside the anomaly detection models. Based on the gradient of the given model, adversarial samples are generated to attack the network. (2) ADS-B black-box attackers cannot obtain the internal structure and parameter information of anomaly detection models and can only attempt to disrupt the behavior of the models by observing ADS-B input data and the output results. In this work, we launch the transfer-based black-box attack against ADS-B anomaly detection models.

**Goals:** Attackers consider two situations: (a) normal to anomaly and (b) anomaly to normal. In (a), adversarial attackers generate the adversarial sample $X^{adv}$ to make the models determine ADS-B normal data as anomaly data incorrectly, hence generating a false-positive. In (b), adversarial attackers create $X^{adv}$ to make the models determine anomaly data as normal data incorrectly, hence generating a false-negative. Adversarial attacks can lead pilots to make incorrect decisions, including changing flight routes, adjusting flight altitude and velocity and even executing emergency landing procedures.

#### 3.4.2. Adversarial Samples Generation

##### Adversarial Samples Generation

The FGSM attack was first proposed in image fields [14]. The FGSM calculates the gradient of the cost function relative to the input of ADS-B anomaly detection models. The FGSM is also known as the one-shot method as adversarial perturbations are generated by a single-step computation. The FGSM generates ADS-B adversarial examples $X^{adv}$ as follows:

(4) $$\left\{ \begin{array}{l} \eta =\varepsilon \times sign(\nabla_{x}J_{f}(X,\;F))\\ X^{adv}=X+\eta \end{array}\right.$$

$J_{f}$ is the cost function of ADS-B anomaly detection model f, $\nabla_{x}$ refers to the gradient of the model f with respect to the original ADS-B time series X with the correct output F, *sign* refers to the sign function, ε is a very small magnitude and $X^{adv}$ is the adversarial sample.

The BIM is a block-based iterative method [15]. The BIM extends the FGSM by applying it multiple times with a small step size and clipping the ADS-B time series after each step. Adversarial samples generated by the BIM are more similar to the original ADS-B time series, which means better stealthiness. This is because the BIM uses a multi-step iterative method with a small step size to generate ADS-B adversarial samples. However, due to the multi-step iterations, the BIM may fall into the local optimum during the process of iterating, resulting in the poor transferability of adversarial attacks. The BIM generates ADS-B adversarial examples $X^{adv}$ as follows:

(5) $$\begin{array}{c} X_{i+1}^{adv}=\min\{X_{i}^{adv}+\varepsilon ,\;\max\{X_{i}^{adv}-\varepsilon ,\;X_{i}^{adv}+\alpha \times sign(\nabla_{x}J_{f}(X_{i}^{adv},\;F))\}\}\\ s.t.\;1\leq i\leq I \end{array}$$

$X_{i}^{adv}$ represents the adversarial sample of the *i*-th iteration. ε represents the amount of maximum perturbation. α represents the per step small perturbation. *sign* refers to the sign function. $\nabla_{x}$ refers to the gradient of the model f with respect to the original ADS-B time series X. $J_{f}$ is the cost function of ADS-B anomaly detection model f. I represents the number of iterations. The three parameters in the BIM usually need to satisfy α×I=ε. The BIM utilizes a min and max clipping function to generate adversarial samples.

##### Our Proposed TNAI-FGSM Generating ADS-B Adversarial Samples

In order to improve the success rate of adversarial attacks, the TNAI-FGSM utilizes a time neighborhood mechanism to craft adversarial ADS-B samples which fully takes into account the temporal correlation of ADS-B data. Additionally, to improve the transferability of adversarial attacks against different ADS-B anomaly detection models, the TNAI-FGSM integrates an accumulation iteration mechanism into the process of adversarial attacks. The accumulation iteration mechanism can stabilize the update directions of adversarial samples and escape from poor local optimum during the process of iterating, resulting in more transferable ADS-B adversarial attacks. By adding the time variable *t*, the TNAI-FGSM generates ADS-B adversarial examples $X^{adv}$ as Equation (6) to Equation (8). Algorithm 1 also specifically shows the process of the TNAI-FGSM generating adversarial samples.

(6) $$E_{L}(X_{i,t})=\frac{1}{2L+1}\sum_{k=-L}^{L}\nabla_{x}J_{f}(X_{i,t+k}^{adv},\;F)$$

(7) $$G_{i+1,t}=G_{i,t}+\frac{E_{L}(X_{i,t})}{{\left\|E_{L}(X_{i,t})\right\|}_{1}}$$

(8) $$X_{i+1,t}^{adv}=X_{i,t}^{adv}+\alpha \times sign(G_{i+1,t})$$

In Equations (6)–(8), *i* represents the *i*-th iteration. *t* denotes ADS-B data at time *t.*

Equation (6) represents the time neighborhood mechanism to craft adversarial ADS-B samples. $\nabla_{x}J_{f}(X_{i,t}^{adv},\;F)$ represents the gradient of the loss function at time *t*. In order to fully take into account the temporal correlation of ADS-B data, $E_{L}(X_{i,t})$ is used to represent the average gradient from time t−L to time t+L. *L* denotes the range of time neighborhood (Section 4.2.3**:** Selection of the range of time neighborhood *L*). The time neighborhood mechanism fully considers the gradient information of the historical and future moments of ADS-B data, and thus generates more stable and reliable adversarial samples with a higher success rate of adversarial attacks.

Equation (7) represents the accumulation iteration mechanism to craft adversarial ADS-B samples. Note that Equation (7) utilizes the accumulation iteration mechanism for crafting adversarial ADS-B samples while considering the time neighborhood mechanism. $G_{i,t}$ represents the cumulative gradient which gathers the gradients of the first *i* iterations at time *t*. In each iteration, the average gradient $E_{L}(X_{i,t})$ is normalized by the *L*_1_ distance (any distance measure is feasible). The accumulation iteration mechanism can memorize the gradients of previous iterations which helps to barrel through narrow valleys, small humps and poor local minima or maxima [38]. We apply the idea to generate ADS-B adversarial examples which can stabilize the update directions of adversarial samples and escape from poor local optimum during the process of iterating, resulting in more transferable ADS-B adversarial attacks. In Equation (8), *sign* refers to the sign function and α represents the per step small perturbation.
**Algorithm 1.** TNAI-FGSM generating ADS-B adversarial samples**Input:**Original ADS-B time series $X=[X_{1},X_{2},\ldots ,X_{t},\ldots ,X_{M}]$ and its anomaly detection result *F*
**Parameters:**The number of iterations *I*, maximum perturbation ε, per step small perturbation α, the range of time neighborhood *L***Output:**ADS-B adversarial sample $X^{adv}$//Initialize adversarial example $X_{0,t}^{adv}$ and cumulative gradient $G_{0,t}$1. $X_{0,t}^{adv}=X_{t},G_{0,t}=0$2. **for**
i=1 to I do//Time neighborhood mechanism calculates the average gradient3.   $E_{L}(X_{i,t})=\frac{1}{2L+1}\sum_{k=-L}^{L}\nabla_{x}J_{f}(X_{i,t+k}^{adv},\;F)$//Accumulation iteration mechanism gathers the gradients of the first *i* iterations4.   $G_{i+1,t}=G_{i,t}+\frac{E_{L}(X_{i,t})}{{\left\|E_{L}(X_{i,t})\right\|}_{1}}$//Calculate the final adversarial example $X_{i+1,t}^{adv}$5.   $X_{i+1,t}^{adv}=X_{i,t}^{adv}+\alpha \times sign(G_{i+1,t})$6. **end for**

### 3.5. Evaluation Index

The confusion matrix for sample classification is given in Table 3. True Positive (TP) indicates that real normal data are detected as normal data. False Negative (FN) indicates that real normal data are detected as anomaly data. False Positive (FP) indicates that real anomaly data are detected as normal data. True Negative (TN) indicates that real anomaly data are detected as anomaly data.

False Positive Rate (FPR), False Negative Rate (FNR), Accuracy and F1_score are used as the evaluation index. FPR, FNR, Accuracy and F1_score are defined as follows:

(9) $$\left\{ \begin{array}{c} \mathrm{FPR}=\frac{\mathrm{FN}}{\mathrm{TP}+\mathrm{FN}}\\ \mathrm{FNR}=\frac{\mathrm{FP}}{\mathrm{FP}+\mathrm{TN}}\\ \mathrm{Accuracy}=\frac{\mathrm{TN}+\mathrm{TP}}{\mathrm{TP}+\mathrm{FN}+\mathrm{FP}+\mathrm{TN}}\\ F1\_\mathrm{score}=\frac{2\mathrm{TP}}{2\mathrm{TP}+\mathrm{FN}+\mathrm{FP}} \end{array}\right.$$

## 4. Experiments

### 4.1. Data Collection

**Figure 3:**
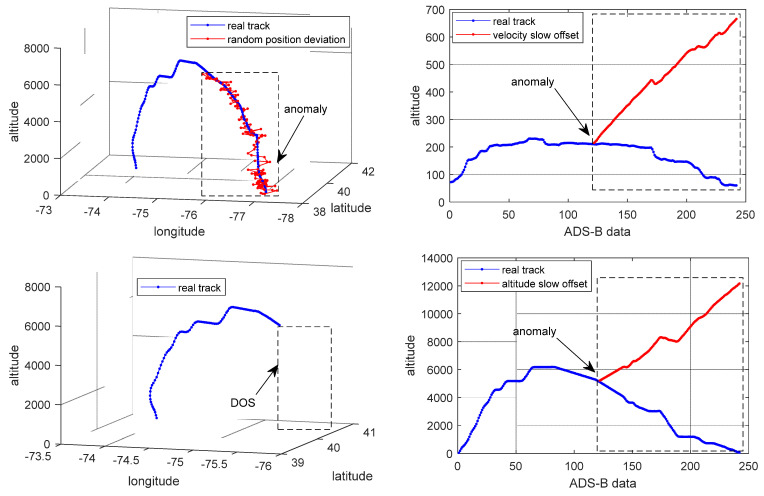
ADS-B anomaly flight track. (The anomaly type of the subgraph in the upper left corner is random position deviation. The upper right corner is velocity slow offset. The lower left corner is DOS. The lower right corner is altitude slow offset).

For the experiment, ADS-B data from 50 flights were collected as training samples from OPENSKY which records ADS-B data from the real world [39]. In addition, ADS-B data from 40 flights were collected as test samples. In OPENSKY, ADS-B data from real world were recorded every 10 s. Each flight includes aircraft’s take-off, climb, cruise, turning and descent phase. Each flight contains ADS-B data from 200 to 1000. Considering the limitations of actual environments, ADS-B anomaly data were difficult to obtain. ADS-B anomaly data in the test dataset were generated by simulation. The simulated anomaly styles include random position deviation, velocity slow offset, Denial of Service (DOS) and altitude slow offset. In order to demonstrate the effect of adversarial attacks, a sample flight containing 242 ADS-B data was selected randomly for testing. Four types of ADS-B anomaly data were simulated based on original ADS-B data which is shown in Table 4 and Figure 3.

### 4.2. Experimental Analysis

#### 4.2.1. Adversarial Attack against VAE

**Figure 4:**
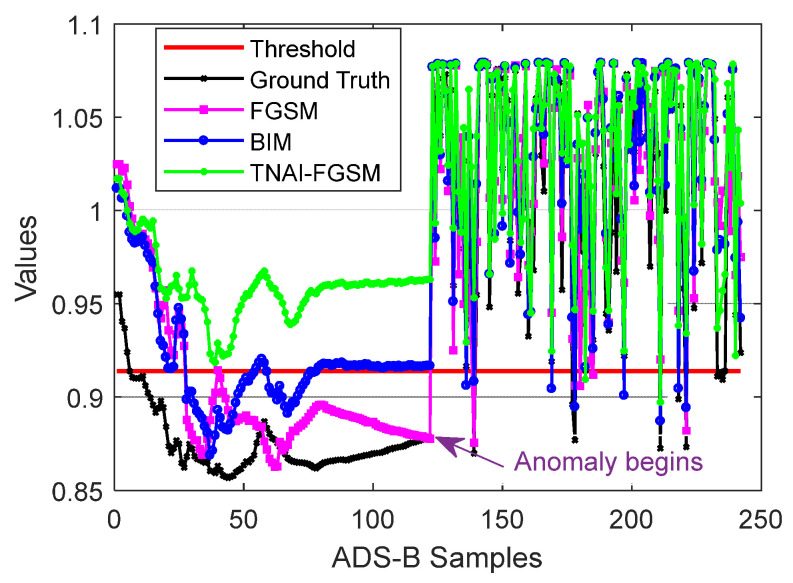
Adversarial attacks against VAE (random position deviation).

We first attacked VAE by using non-targeted FGSM, BIM and TNAI-FGSM with the size of adversarial perturbation ε=0.05, the per step small perturbation α=0.001, the number of iterations I=50, the range of time neighborhood L=6 (Section 4.2.3**:** Selection of the range of time neighborhood *L*). In order to demonstrate the effect of adversarial attacks, a sample flight containing 242 ADS-B data was selected randomly for testing. The first half of ADS-B data contained the aircraft’s take-off, climb, turning and cruise phase. The last half of ADS-B data contained the aircraft’s cruise, turning and descent phase. The simulated ADS-B anomaly data began in the last half of the ADS-B data. When the anomaly type of ADS-B data was random position deviation, the result of adversarial attacks against VAE is depicted in Figure 4. We can analyze Figure 4 to obtain the following results. **For ground truth (no adversarial attacks**), in the first half of normal test samples, there were 5 samples whose anomaly values were greater than the threshold, thus FPR was 4.13%. In the last half of anomaly test samples, there were 9 samples whose anomaly values were less than the threshold, thus FNR was 7.44%. Accuracy was 94.22%. F1_score was 94.31%. **For FGSM adversarial attack**, in the first half of normal test samples, there were 29 samples whose anomaly values were greater than the threshold, thus FPR was 23.97%. In the last half of anomaly test samples, there were 8 samples whose anomaly values were less than the threshold, thus FNR was 6.61%. Accuracy was 84.71%. F1_score was 83.26%. **For BIM adversarial attack**, in the first half of normal test samples, there were 74 samples whose anomaly values were greater than the threshold, thus FPR was 61.16%. In the last half of anomaly test samples, there were 7 samples whose anomaly values were less than the threshold, thus FNR was 5.79%. Accuracy was 66.53%. F1_score was 53.71%. **For TNAI-FGSM adversarial attack**, in the first half of normal test samples, there were 121 samples whose anomaly values were greater than the threshold, thus FPR was 100%. In the last half of anomaly test samples, there are 2 samples whose anomaly values were less than the threshold, thus FNR was 1.65%. Accuracy was 49.17%. F1_score was 0%.

**Figure 5:**
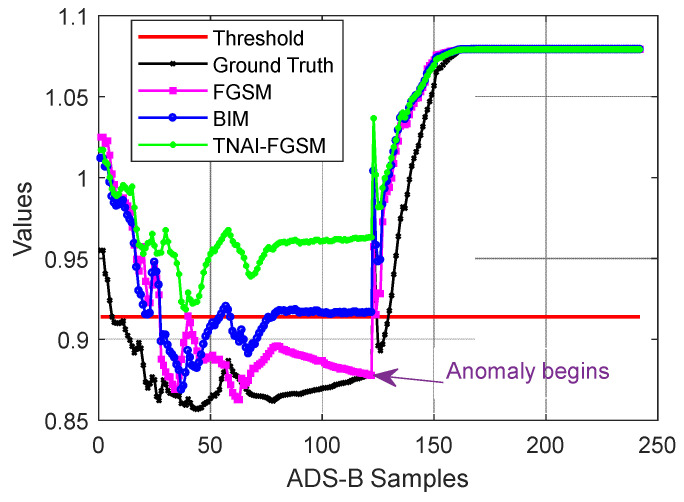
Adversarial attacks against VAE (velocity slow offset).

**Figure 6:**
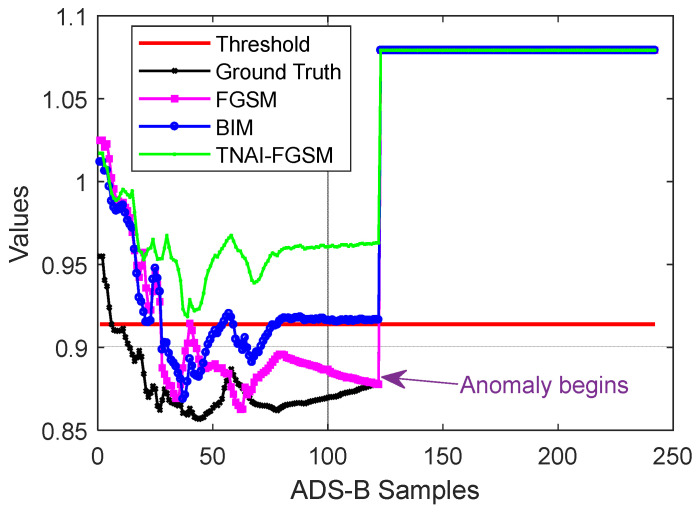
Adversarial attacks against VAE (DOS).

**Figure 7:**
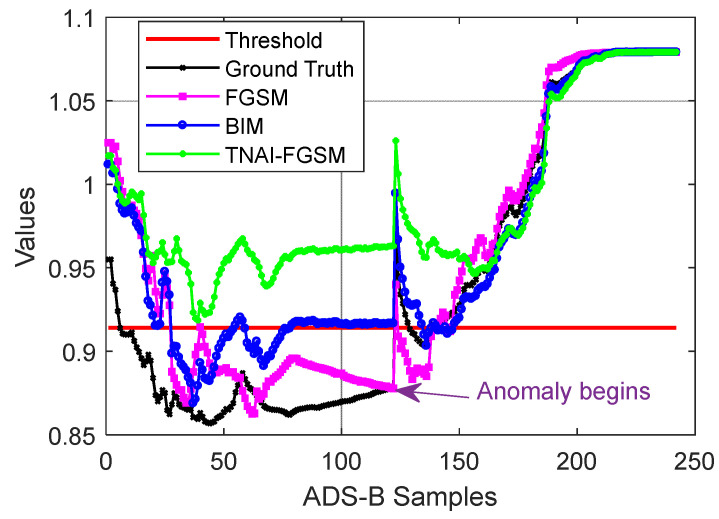
Adversarial attacks against VAE (altitude slow offset).

When the anomaly type of ADS-B data was velocity slow offset, DOS and altitude slow offset, the results of adversarial attacks against VAE are depicted from Figure 5, Figure 6 and Figure 7. The experiments tested 40 flights for adversarial attacks against VAE model, and the average values of FPR, FNR, Accuracy and F1_score of the 40 flights with four types of anomaly ADS-B data were taken as the experimental results. Table 5 shows the experimental results of adversarial attacks against VAE model. By analyzing Table 5, we can draw the following conclusions. (1) For FPR, a small, imperceptible adversarial perturbation can result in VAE model producing a false alarm, with TNAI-FGSM performing the best. In other words, TNAI-FGSM adversarial attack makes VAE model incorrectly interpret over 90% of ADS-B normal data as anomaly data. (2) For FNR, adversarial attacks helped VAE models detect ADS-B anomaly data. It is an interesting phenomenon that adversarial attacks against unsupervised anomaly detection models can lead to a decrease in FNR. This may be because adversarial attacks can increase the degree of anomaly in ADS-B data, making ADS-B anomaly data easier to detect. (3) For Accuracy and F1_score, TNAI-FGSM adversarial attack performed best. In other words, TNAI-FGSM is better than FGSM and BIM in fooling ADS-B anomaly detection models. The reason is that TNAI-FGSM improved FGSM and BIM by utilizing time neighborhood mechanism and accumulation iteration mechanism, which resulted in a better attack impact.

**Figure 8:**
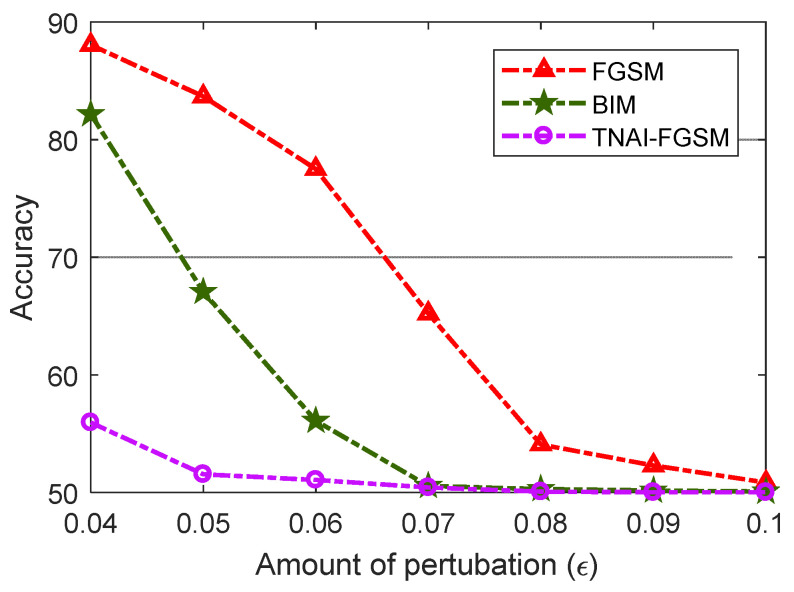
Accuracy variation with respect to the amounts of perturbations.

In Figure 8, we evaluated VAE model’s Accuracy with respect to the different amounts of perturbations. By analyzing Figure 8, we can draw the following conclusions. (1) For FGSM adversarial attack, larger values of ε made Accuracy gradually decrease. When ε was greater than 0.08, Accuracy was not significantly decreased. For BIM adversarial attack, when ε was greater than 0.07, Accuracy was not significantly decreased. For TNAI-FGSM adversarial attack, when ε was greater than 0.05, Accuracy was not significantly decreased. The results can provide guidance for attackers when selecting attack parameters. (2) We observed that for the larger value of ε, Accuracy gradually decreased and converged to around 50%. This was due to normal and anomaly data; each accounted for half of the ADS-B test data in this experiment. Adversarial attack makes VAE model incorrectly interpret almost all of ADS-B normal data as anomaly data with the larger value of ε. However, adversarial attacks against VAE model can gradually reduce FNR almost to 0 with the larger value of ε.

#### 4.2.2. Adversarial Attack against LSTM, GRU and LSTM-Encoder-Decoder

**Figure 9:**
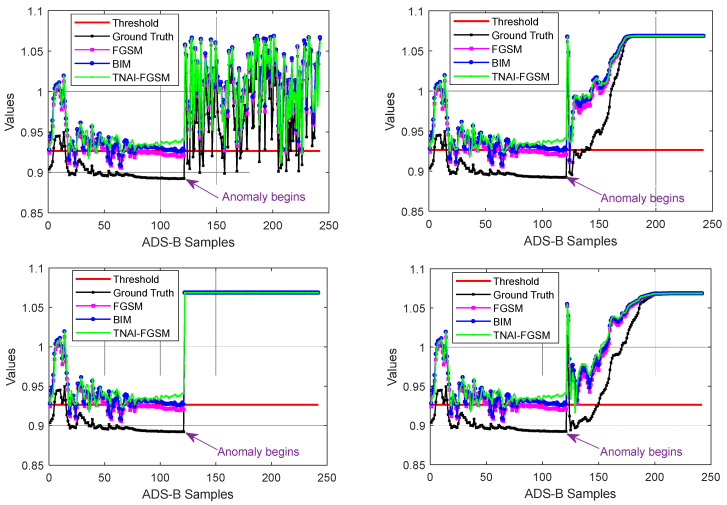
Adversarial attacks against LSTM. (The anomaly type of the subgraph in the upper left corner is random position deviation. The upper right corner is velocity slow offset. The lower left corner is DOS. The lower right corner is altitude slow offset).

**Figure 10:**
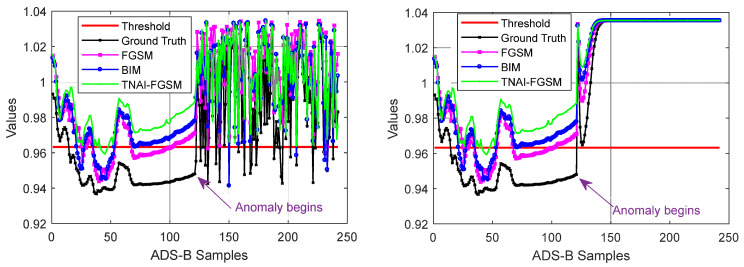

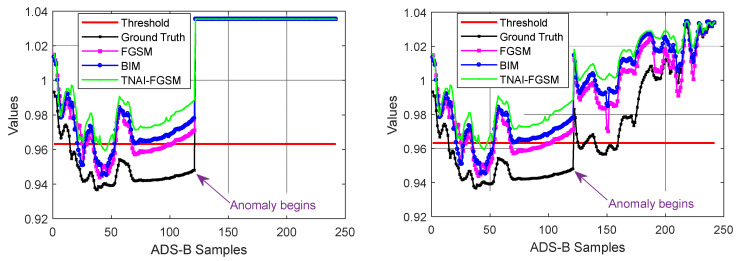
Adversarial attacks against GRU. (The anomaly type of the subgraph in the upper left corner is random position deviation. The upper right corner is velocity slow offset. The lower left corner is DOS. The lower right corner is altitude slow offset).

**Figure 11:**
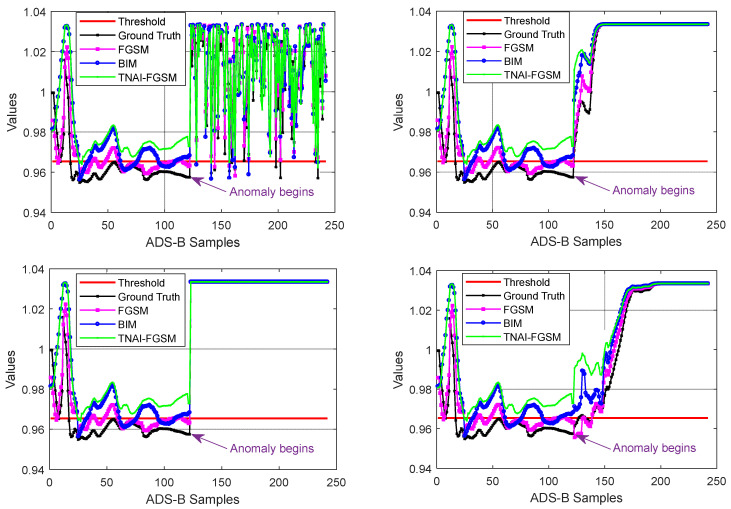
Adversarial attacks against LSTM-Encoder-Decoder. (The anomaly type of the subgraph in the upper left corner is random position deviation. The upper right corner is velocity slow offset. The lower left corner is DOS. The lower right corner is altitude slow offset).

As with LSTM, GRU and LSTM-Encoder-Decoder, we first used non-targeted FGSM, BIM and TNAI-FGSM with the size of adversarial perturbation ε=0.05, the per step small perturbation α=0.001, the number of iterations I=50 and the range of time neighborhood L=6 (Section 4.2.3**:** Selection of the range of time neighborhood *L*). A sample flight containing 242 ADS-B data was selected randomly, which was the same as VAE. When the anomaly type of ADS-B data was random position deviation, velocity slow offset, DOS and altitude slow offset, the results of adversarial attacks against LSTM, GRU and LSTM-Encoder-Decoder are depicted from Figure 9, Figure 10 and Figure 11. Table 6 shows the average FPR, FNR, Accuracy and F1_score results of adversarial attacks against LSTM, GRU and LSTM-Encoder-Decoder model. By analyzing Table 6, we can draw the following conclusions. For FPR, a small imperceptible adversarial perturbation can result in LSTM, GRU and LSTM-Encoder-Decoder model to produce false alarm, with TNAI-FGSM performing best. For FNR, adversarial attacks helped LSTM, GRU and LSTM-Encoder-Decoder model detect ADS-B anomaly data. For Accuracy and F1_score, TNAI-FGSM adversarial attack performed best with the size of adversarial perturbation ε=0.05. In other words, TNAI-FGSM was better than FGSM and BIM in fooling ADS-B anomaly detection models when the attack was stealthy. The reason was that TNAI-FGSM improved FGSM and BIM by utilizing time neighborhood mechanism and accumulation iteration mechanism, which resulted in a better attack impact.

**Figure 12:**
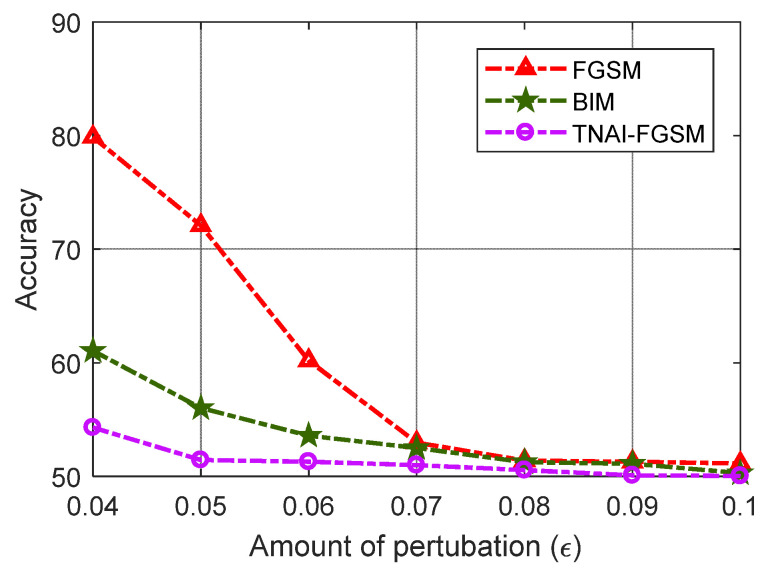
LSTM Accuracy variation with respect to the amounts of perturbations.

**Figure 13:**
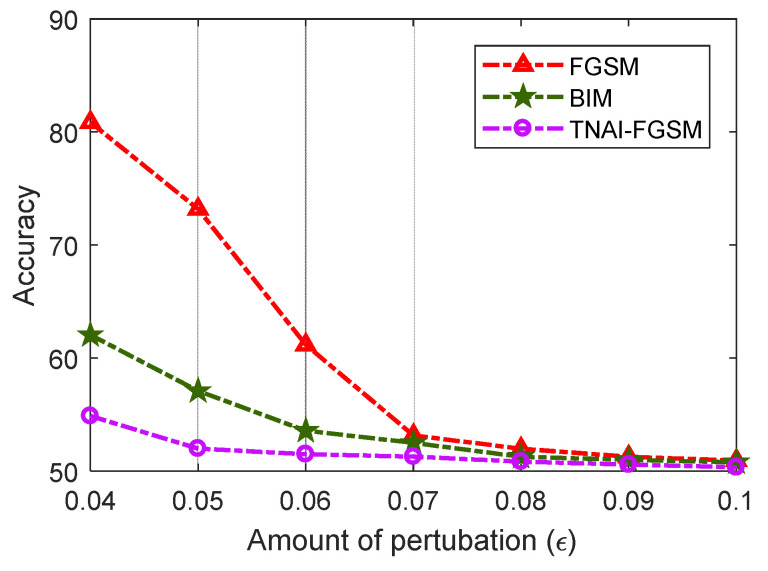
GRU Accuracy variation with respect to the amounts of perturbations.

**Figure 14:**
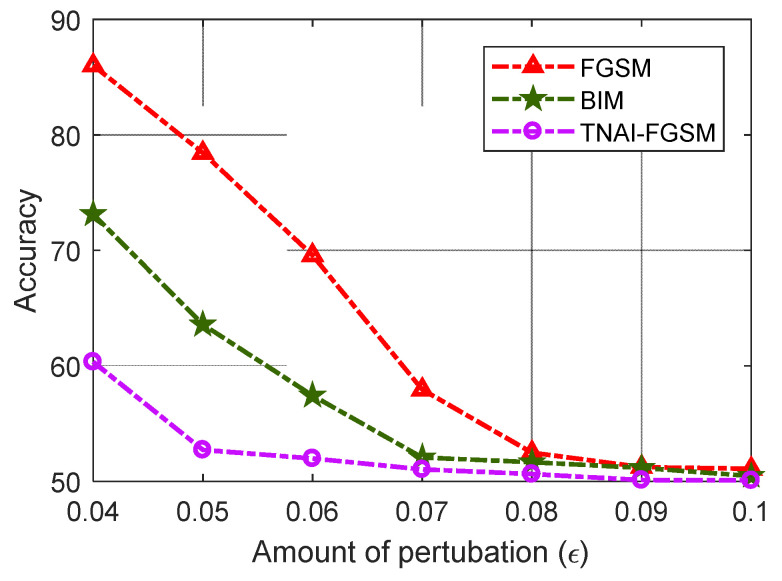
LSTM-Encoder-Decoder Accuracy variation with respect to the amounts of perturbations.

From Figure 12, Figure 13 and Figure 14, we evaluated LSTM, GRU and LSTM-Encoder-Decoder model’s Accuracy with respect to the different amounts of perturbations allowed for crafting the adversarial ADS-B data. We can draw the following conclusions. (1) For FGSM, BIM and TNAI-FGSM adversarial attacks, the larger value of ε made Accuracy gradually decrease and converge to around 50%. (2) For FGSM attack, Accuracy of LSTM and GRU was not significantly decreased when ε was greater than 0.07. However, Accuracy of LSTM-Encoder-Decoder was not significantly decreased when ε was greater than 0.08. (3) For BIM attack, Accuracy of LSTM and GRU were not significantly decreased when ε was greater than 0.06. However, Accuracy of LSTM-Encoder-Decoder was not significantly decreased when ε was greater than 0.07. The results can provide guidance for attackers when selecting attack parameters. (4) The results also showed that TNAI-FGSM was better than FGSM and BIM in fooling ADS-B anomaly detection models when the attack was stealthy. TNAI-FGSM decreased Accuracy more when compared to FGSM and BIM especially when the attack was stealthy with ε not greater than 0.05. The reason was that TNAI-FGSM improved FGSM and BIM by utilizing time neighborhood mechanism and accumulation iteration mechanism, which resulted in a better attack impact.

In the experiments, the average time spent by TNAI-FGSM in generating ADS-B adversarial samples was recorded. TNAI-FGSM required 5.63 s to generate 100 ADS-B adversarial samples. ADS-B data was broadcast once per second on average. Therefore, TNAI-FGSM generated ADS-B adversarial samples in real-time compared to the frequency at which ADS-B data was broadcast. It proved that TNAI-FGSM was efficient in generating ADS-B adversarial samples.

#### 4.2.3. Selection of the Range of Time Neighborhood L

**Figure 15:**
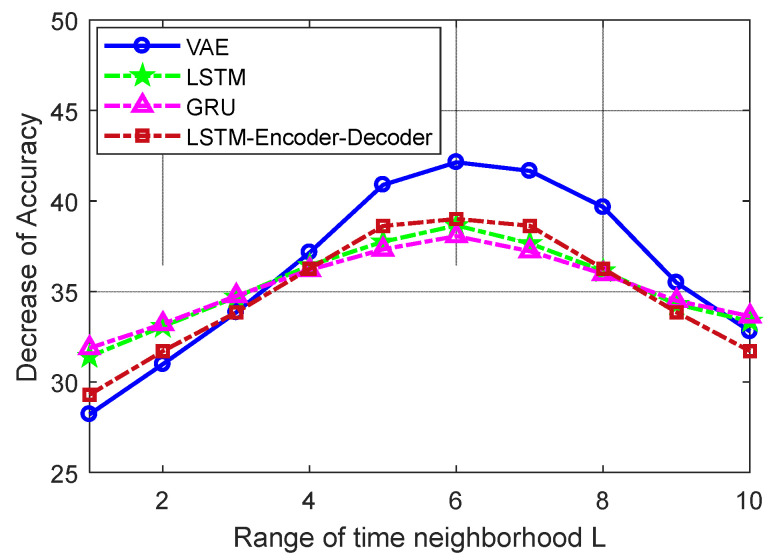
Selection of the range of time neighborhood.

In order to fully utilize temporal correlation of ADS-B data to craft adversarial samples, TNAI-FGSM needed to choose an appropriate range of time neighborhood *L*. If the range of time neighborhood is too short, TNAI-FGSM will lose a large amount of valid gradient information coming from time neighborhood, resulting in the poor performance of adversarial attacks. If the range of time neighborhood is too long, TNAI-FGSM will memorize too much invalid gradient information, resulting in the poor performance of adversarial attacks as well. In order to obtain an appropriate range of time neighborhood, this paper compares the decrease in Accuracy of anomaly detection under different ranges of time neighborhood. As shown in Figure 15, when the range of time neighborhood gradually increased to 6, the decrease in Accuracy of anomaly detection reached the optimum. When the range of time neighborhood exceeded 6, the decrease in Accuracy deteriorated. Therefore, an appropriate range of time neighborhood was selected as 6.

#### 4.2.4. Transfer-Based Black-Box Attack

To evaluate the transferability of ADS-B adversarial attacks, we applied ADS-B adversarial examples crafted for LSTM model on the GRU, LSTM-Encoder-Decoder and VAE model. The size of adversarial perturbation we set was ε=0.07. Under this parameter setting, decrease in Accuracy of LSTM converged. Table 7 summarizes the obtained results on transferability. We observed that for all the GRU, LSTM-Encoder-Decoder and VAE models, ADS-B adversarial examples crafted for LSTM were transferable. For instance, ADS-B adversarial examples crafted using FGSM for LSTM model caused an 18.63, 17.41% and 14.24% decrease when transferred to the GRU, LSTM-Encoder-Decoder and VAE model. A similar trend was also observed, however, with a larger percentage increase when ADS-B adversarial examples crafted using BIM and TNAI-FGSM for LSTM model were transferred to GRU, LSTM-Encoder-Decoder and VAE model.

In addition, the results also showed that our proposed TNAI-FGSM was better than FGSM and BIM in fooling ADS-B anomaly detection models. TNAI-FGSM decreased Accuracy more when compared to the FGSM and BIM. The reason was that TNAI-FGSM integrated accumulation iteration mechanism into the process of adversarial attacks. Accumulation iteration mechanism can stabilize the update directions of adversarial samples and escape from poor local optimum during the process of iterating, resulting in more transferable ADS-B adversarial attacks.

Overall, the results showed that ADS-B adversarial examples can be transferred to different ADS-B anomaly detection models. This type of attack is known as transfer-based black-box attack, where attackers do not have access to the target model’s internal parameters, yet they are able to generate perturbed ADS-B time series that can fool ADS-B anomaly detection models.

#### 4.2.5. Defense against Adversarial Attacks

The existing adversarial attack defense approaches applied in computer vision areas mainly include blocking the transferability [40], gradient hiding [41], defensive distillation [42] and adversarial training [14]. We analyzed the applicability of each defense method for ADS-B adversarial attacks.

(1) Blocking the transferability: To block the transferability, this method was proposed such that, as the input is more perturbed, the classifier smoothly outputs lower confidence on the original label and instead predicts that the input is invalid. In essence, this method augments the output class set with a NULL label and trains the classifier to reject the adversarial examples by classifying them as NULL. However, blocking the transferability required the dataset to be labeled, thus it was not applicable to unsupervised machine learning including deep-learning-based ADS-B unsupervised anomaly detection.

(2) Gradient hiding: This method constructed a model that did not have useful gradients, e.g., by using a nearest neighbor classifier instead of a Deep Neural Network (DNN). This method made it difficult to construct adversarial examples due to the absence of a gradient. However, it was not applicable to deep-learning-based ADS-B unsupervised anomaly detection models which are based on a DNN with the presence of the gradients.

(3) Defensive distillation: This method trained a DNN-based classifier that was more robust to input perturbations. Defensive distillation extracted additional knowledge about training points as class probability vectors produced by a DNN, which was fed back into the training regimen. To defend against such perturbations, defensive distillation attempts to reduce variations around the input, and consequently the amplitude of adversarial gradients. In other words, defensive distillation smooths the model learned by a DNN architecture during training by helping the classifier generalize better to samples outside of its training dataset. However, defensive distillation required the dataset to be labeled, thus it was not applicable to unsupervised machine learning including deep-learning-based ADS-B unsupervised anomaly detection.

(4) Adversarial training: The core idea of adversarial training is to use ADS-B adversarial samples as additional training data so that ADS-B anomaly detection models are more robust to adversarial perturbations. Adversarial training can be performed without the need for the training dataset to be labeled, thus it was suitable for ADS-B anomaly detection. Table 8 shows the accuracy performance of the four deep-learning-based anomaly detection models before adversarial attacks, after adversarial attacks and after adversarial training. For adversarial training, 20 of the 40 test flights processed by adversarial attacks were added to the training dataset, and the remaining 20 test flights were used as the test dataset. The results in Table 8 were obtained with the setting of adversarial perturbation ε=0.05, the per step small perturbation α=0.001, the number of iterations I=50 and the range of time neighborhood L=6. As can be seen in Table 8, Accuracy of LSTM, GRU, LSTM-Encoder-Decoder and VAE were restored to more than 75% after adversarial training. The results verified that adversarial training is a generalized defense strategy. In other words, adversarial training is effective for the security defense of different deep-learning-based ADS-B unsupervised anomaly detection models suffering from different adversarial attacks.

## 5. Limitations and Future Work

This work verifies that ADS-B anomaly detection models are vulnerable to adversarial attacks and ADS-B adversarial attacks have the characteristic of transferability. However, there may be some limitations in our work, and future work will try to solve them.

(1) This work assumes that the transfer-based black-box attack can fool other ADS-B anomaly detection models. However, except for the VAE, LSTM, GRU and LSTM-Encoder-Decoder, other anomaly detection models which are based on Transformer [43] and Graph Neural Network (GNN) [44] have not been experimentally verified to determine to what extent they are prone to adversarial attacks. We can address this issue in two ways in the future. First, we could consider more efficient and general black-box adversarial attacks. Second, we can use other anomaly detection models which are based on Transformer and GNN as the victim models for experiments.

(2) Although we launched the transfer-based black-box attacks against ADS-B anomaly detection models, the decrease in accuracy was not the most efficient. To address this issue, we will further explore other advanced black-box attack methods including a query-based attack [45] and data-free black-box adversarial attack [46]. When considering a query-based attack against ADS-B anomaly detection models, the most important thing is how to reduce the number of queries and thus reduce the complexity. When considering data-free black-box adversarial attacks against ADS-B anomaly detection models, the most important thing is how to design an efficient Generative Adversarial Network (GAN) for obtaining the substitute model.

(3) For the security defense against ADS-B adversarial attacks, this work explores and discusses defense mechanisms including blocking the transferability, gradient hiding, defensive distillation and adversarial training. Blocking the transferability requires the dataset to be labeled, thus it is not applicable to unsupervised machine learning including deep-learning-based ADS-B unsupervised anomaly detection. Gradient hiding is applicable to the model that does not have useful gradients. Gradient hiding is applicable to the models without gradients, thus it is not suitable for ADS-B anomaly detection models which are based on a DNN with the presence of the gradients. Defensive distillation requires the dataset to be labeled, thus it is not applicable to unsupervised machine learning including deep-learning-based ADS-B unsupervised anomaly detection. Adversarial training can be performed without the need for the training dataset to be labeled, thus it can be used for ADS-B anomaly detection. However, adversarial samples are able to evolve continuously by adversarial attackers, thus adversarial training cannot solve the evolved adversarial samples. Due to the increasing threat of adversarial machine learning to deep-learning-based ADS-B unsupervised anomaly detection models, we will thus improve the robustness of ADS-B anomaly detection models from their initial designs which consider adversarial attacks in the future.

## 6. Conclusions

In this work, the concept of adversarial attacks against deep-learning-based ADS-B unsupervised anomaly detection models is considered. We define and formalize adversarial attacks against deep-learning-based ADS-B time series unsupervised anomaly detection models. We propose the TNAI-FGSM for adversarial sample generation which is based on the time neighborhood mechanism and accumulation iteration mechanism. The obtained results show the impact of adversarial samples on four types of ADS-B unsupervised anomaly detection models. The results also show that the TNAI-FGSM is better than the FGSM and BIM in fooling ADS-B anomaly detection models when the attack is stealthy. Additionally, ADS-B adversarial examples can be transferred to different ADS-B anomaly detection models. Furthermore, adversarial training is used to defend against ADS-B adversarial attacks. Our study differs from previous research because we focus on adversarial attacks against ADS-B time series unsupervised anomaly detection rather than the broader classification problem. Through our work, we want to raise awareness of the adversarial vulnerability of ADS-B anomaly detection models which is vital in safety-critical and cost-critical ATM. In the future, we plan to extend our work by utilizing other adversarial attacks against ADS-B anomaly detection models and investigate more defense strategies to mitigate ADS-B adversarial attacks.

## Figures and Tables

**Table 1:** Comparison of related ADS-B solutions.

Methods		Protocol Modification Required or Additional Nodes Required	Advantages and Disadvantages
Traditional methods	Encryption	Require modifying original ADS-B protocol	Disadvantages: Encryption method needs to modify the original ADS-B protocol, which hinders system compatibility
	Physical layer information	Require ground stations and other entities	Disadvantages: When attackers obtain prior knowledge of physical layer information through statistical analysis, the applicability of the method will need further verification for sophisticated ADS-B data attacks
	Multilateration	Require more than one sensor or entity	Disadvantages: Multilateration method requires deploying multiple ground stations, which is not economical
Deep-learning-based anomaly detection		Do not require modifying protocol and additional nodes	Advantages: Deep learning method makes full use of ADS-B time series to detect anomaly data quickly and accurately

**Table 2:** Gaps of previous methods.

Name of the Method	Gaps When the Methods Are Considered to Be Applied to ADS-B Anomaly Detection Models
MEA [24]	The biggest flaw is that MEA assumes that the victim models are supervised and assumes ADS-B spoofing detection is a classification problem. However, there is scarce labeled ADS-B anomaly data in a real-world environment.
FGSM [14,35] and BIM [15,36]	FGSM and BIM fail to take into account temporal correlation of ADS-B time series. Also, they cannot stabilize the update directions of adversarial samples and cannot escape from poor local optimum during the process of iterating.

**Table 3:** Confusion matrix for sample classification.

Confusion Matrix	Detected Normal Data	Detected Anomaly Data
Real normal data	TP	FN
Real anomaly data	FP	TN

**Table 4:** Experiment dataset.

Anomaly Types	Data Simulated Methods
Random position deviation	Inject Gaussian noise with a mean value 0 and a variance 0.5 into the actual latitude and longitude
Velocity slow offset	Enlarge the velocity with a multiple of 5 gradually
DOS	The aircraft track disappears in the air traffic surveillance system
Altitude slow offset	Enlarge the altitude with a multiple of 100 gradually

**Table 5:** Adversarial attack performance against VAE (%).

Evaluation Index	No Adversarial Attack	FGSM	BIM	TNAI-FGSM
FPR	4.93	25.75	60.59	91.05
FNR	7.71	6.83	5.29	2.44
Accuracy	93.69	83.68	67.06	51.55
F1_score	93.79	81.99	54.47	13.59

**Table 6:** Adversarial attack performance against LSTM, GRU and LSTM-Encoder-Decoder (%).

	Evaluation Index	No Adversarial Attacks	FGSM	BIM	TNAI-FGSM
**LSTM**	FPR	9.08	52.13	85.11	94.46
	FNR	10.73	3.71	2.85	2.73
	Accuracy	90.09	72.08	56.02	51.44
	F1_score	90.17	63.15	25.29	10.29
**GRU**	FPR	9.02	50.32	83.35	92.75
	FNR	10.89	3.84	2.93	2.82
	Accuracy	90.03	73.17	57.09	51.97
	F1_score	90.11	64.24	26.27	11.25
**LSTM-Encoder-Decoder**	FPR	7.91	35.99	67.35	90.95
	FNR	8.60	7.14	5.48	3.55
	Accuracy	91.75	78.44	63.59	52.74
	F1_score	91.77	74.82	47.27	16.11

**Table 7:** Transferability of ADS-B adversarial attacks (% decrease in Accuracy).

	GRU	LSTM-Encoder-Decoder	VAE
FGSM (LSTM)	18.63	17.41	14.24
BIM (LSTM)	23.54	21.74	17.48
TNAI-FGSM (LSTM)	36.87	35.38	28.53

**Table 8:** Accuracy performance of ADS-B anomaly detection models (%).

	LSTM	GRU	LSTM-Encoder-Decoder	VAE
Before adversarial attacks	90.09	90.03	91.75	93.69
After FGSM attacks	72.08	73.17	78.44	83.68
After adversarial training	84.24	85.13	86.95	88.75
After BIM attacks	56.02	57.09	63.59	67.06
After adversarial training	82.58	82.83	83.32	83.09
After TNAI-FGSM attacks	51.44	51.97	52.74	51.55
After adversarial training	75.43	77.85	78.23	77.34

## Data Availability

The original contributions presented in the study are included in the article, further inquiries can be directed to the corresponding author.
